# Addenbrooke's Hospital, Cambridge

**Published:** 1892-07-16

**Authors:** 


### The Institutional Workshop.

### WITHIN THE HOSPITALS.

### V.—ADDENBROOKE'S HOSPITAL, CAMBRIDGE.

The plan on which this hospital has been constructed, so far as the main buildings are concerned, is known as the straight pavilion plan. The administration block is situated in the centre, and on either side are two pavilion wards which on the upper floors contain nineteen beds each. They were erected in 1864, and it is interesting to note that, whereas the architect, Sir M. D. P. Wyatt, provided at one end and side that the beds should have windows between every two beds for purposes of air and light, the wards contain linked beds at one end where this provision has been ignored. Experience shows that those beds in a pavilion ward which are placed in the corners, without a window on each side, invariably prove to be those the occupants of which are most liable to tonsilitis and kindred troubles, including unhealthy wounds in surgical cases. Since 1864, linked beds for hospital wards constructed for the accommodation of acute cases have been given up and each bed is now provided, in all the well-constructed and modern hospitals, with windows on each side of it in addition to an abundance of floor space.

#### The Wards.

The wards at Addenbrooke's Hospital, having regard to the date of construction are satisfactory, the amount of cubic space is ample, and there is an abundance of light in all except the accident ward on the ground floor. We think they would be improved by the introduction of ventilators, as the air struck us as being somewhat heavy in some of the wards. There are central fireplaces, the flues of which are so arranged as to add to the appearance whilst providing for free circulation of air. The floors, which are unpolished, showed considerable evidence of wear and tear, and will probably have to be renewed in a few years.

#### The Out-Patient Department.

This is situated beneath the main medical ward, and consists of an out-patient waiting hall of ample proportions, physicians' and surgeons' consulting-rooms, a dentist's room, a dispensary, and other offices. Out-patient practice is encouraged at Addenbrooke's Hospital, owing, no doubt, to the important medical school which is attached to it, and on the day of our visit, a Saturday, three hundred out-patients had been under treatment.

#### The Drainage.

Addenbrooke's Hospital, from the date of its reconstruction, has laboured under the misfortune of an old drainage system which has proved to be inadequate and unsatisfactory. A brick sewer runs underneath the accident ward on the one side, and one of the main drains is similarly situated on the other. The baths—Rufford's—are in good order, although they have been in use for nearly thirty years, but the w.c.'s are of an old pattern, and there are practically no slop-sinks. In these circumstances it is satisfactory to know that Mr. Rogers Field has been called in to overhaul the whole of the drainage, and we are sure the committee will be well advised in connection with any reconstruction of the present system to supply modern slop-sinks of the very best pattern, new water-closets, and new porcelain fittings in the ward sculleries, as well as in the lavatory and w.c. sections of the building.

#### Site and Out-lying Buildings.

The site of the hospital is self-contained, being bounded on one side by the Tennis Court Road, and on the other by the main road. There are ample lawns in front, and behind is a garden containing trees, which make it very attractive and pleasant. The Matron's sitting-room is a delightful apartment, opening into the garden, and the same may be said of the nurses' dining-room, and of some of the other rooms devoted to the officers. Behind the pavilion block, in which the out-patient department is situated, are the children's wards, an eye-room, and an isolation-ward, with beds. There is also an extensive wash-house and a mortuary. The Nursing Home is situated in one corner of the gardens, to which a new wing has been recently added, as a memorial to the late Miss Alice Fisher, who originated trained nursing at this institution. The Nursing Home contains cubicles, but the architect has made them unnecessarily small, and has unfortunately omitted to provide that each cubicle shall have a separate window, and a full amount of floor space. It therefore happens that although the Nursing Home was specially erected, the cubicles will not compare favourably with those of the Portsmouth Hospital, although the latter have been made out of old wards converted to their present use. We hope the time has gone by when any architect who may be charged with the responsibility of erecting a nursing home in connection with a public institution, will omit to provide that each cubicle shall contain adequate floor space, and a separate window and fire-place as well.

#### Children's Wards.

There are two children's wards presided over by one Sister, and the condition of these wards and the happy faces of the children prove that she is a very able woman, and well up to her difficult duties. It is recorded of her that she objects, and we think rightly objects, to any child being admitted to one of the general wards, as she considers the sick children of Cambridgeshire to be her own particular charge. The impression that the Sister gave us was so favourable, as to recall the good order, splendid discipline, and comfort, which Sister Queen maintained at the London Hospital in her day. The children's department of Addenbrooke's leaves little to be desired, though it struck us that in one of the wards there were too many cots. We understood that they are seldom, if ever, all occupied, but at the same time our experience leads us to think it undesirable to have more cots in a children's ward than is warranted by the superficial and cubic space of such ward. The approach to the children's ward was made attractive by flowers. There is a toy-room, so that the children are taught to value and appreciate their playthings, and a visit to this part of the hospital must rejoice the heart of anyone who has had experience of the working of medical institutions.

#### General Considerations.

No one can visit Addenbrooke's Hospital without being struck with its homeliness and comfort. These attributes impress the visitor and prove that those who are responsible for the daily working of the institution take a great personal interest in the welfare and comfort of the patients committed to their charge.

At the last meeting of the Governors of the Radcliffe Infirmary, Dr. Collier is reported to have said that he knew Addenbrooke's Hospital very well; he had resided in it and was a life governor, and he was quite certain of this, that the sanitary condition of the Radcliffe Infirmary was quite as good, if not better, than that of Addenbrooke's Hospital. We have often pointed out that we were not seriously impugning the sanitary condition of the Radcliffe Infirmary; but we do maintain that Addenbrooke's Hospital is a palace compared with the old block of the Radcliffe Infirmary, which was condemned by Howard, and which we venture to say will not be allowed to remain standing another year at Oxford if its inadequacy and the impression it makes upon a visitor can be driven home to the consciences of the people of the City, University, and County of Oxford. Again, Addenbrooke's is a great medical school—more than forty students were under examination in its wards shortly before we went over it—its medical staff are eminent, alive, and efficient to the fullest extent, and in all these respects Cambridge sets Oxford an example it should not be slow to follow if its residents possess one spark of self-respect and a due regard to its well-being and reputation. Speaking generally, we may say, now that the drainage at Addenbrooke's is to be seen to by Mr. Rogers Field, that the whole of the institution is satisfactory and well up to modern requirements. Its Committee seem, however, to be indifferent to outward appearances, or we are at a loss to account for the fact that the corridors and some of the wards have been left far too long without a coat of paint. This indifference is apparent, too, in the exposure of the coal-bunks on the landings, and the absence of suitable furniture in the balconies for the patients' use. We commend the present condition of these balconies to the attention of the ladies of Cambridge, and we would urge them to take the matter in hand, to supply both boxes to contain flowers, and proper and comfortable seats for the patients, so that the fullest advantage may be taken of the balconies in fine weather by the inmates of the hospital. No doubt these are relatively minor points, but they are well worthy of the Committee's attention, and we shall hope to see an improvement in the directions indicated, especially as the cost of doing the whole of what is necessary would, in reality, prove a mere trifle.

### Special Features.

The history of the Samaritan Fund at Addenbrooke's is interesting, and its system of administration probably unique. It was started in 1806, with a donation of twenty guineas "by an individual on being releassd from a bed of sickness." This money was invested and subsequently added to until the invested property of the fund amounted to nearly £1,000. The interest upon this money, so far as may be necessary, is expended by the Matron, whose personal communication with the patients enables her to become acquainted with the circumstances of each one. In cases of necessity she applies to the weekly board when the patient leaves the hospital, and so provides for the aftercare of such cases, thereby securing the speedy recovery of the breadwinner, to the no small advantage of his family, and may we not say of the country also.

### The Nursing.

We have dealt with the nursing in a separate article in *The Mirror*, but this report would be incomplete were we not to offer our congratulations to the Committee, who now possess in the Sisters and Head Nurses a trained body of ladies whose presence must prove of the greatest benefit to the patients. Indeed, the condition of the wards, their brightness, the air of comfort which they present, and the general efficiency and neatness which characterises the arrangements throughout each Sister's department, eloquently testify to the success of a system which was introduced originally by the late Miss Fisher, and which is another proof of her foresight, courage, and competence. Each Sister considers her ward as a part of herself, and it is her pride and pleasure to strive to make it the most attractive and best administered portion of the institution with which she is proud to be connected. We believe that all the Sisters, and also the present Matron, Miss Cureton, were trained at Addenbrooke's, and it was a pleasure to meet them and to see the genuine interest they took in their work, and the excellent results which such devoted service has secured to the poor of Cambridge and the neighbourhood.

